# Cross-cultural adaptation and validity of the Spanish central sensitization inventory

**DOI:** 10.1186/s40064-016-3515-4

**Published:** 2016-10-21

**Authors:** Antonio Ignacio Cuesta-Vargas, Cristina Roldan-Jimenez, Randy Neblett, Robert J. Gatchel

**Affiliations:** 1 Departamento de Fisioterapia, Cátedra de Fisioterapia, Instituto Investigación de Biomédica de Málaga (IBIMA) Av/Arquitecto Peñalosa, Universidad de MálagaAndalucía Tech (Teatinos Campus Expansión), 29071 Málaga, Spain; 2 Faculty of Health, School of Clinical Science, Queensland University of Technology, Brisbane, QLD Australia; 3 PRIDE Research Foundation, Dallas, TX USA; 4 Department of Psychology, College of Science, Center of Excellence for the Study of Health and Chronic Illnesses, The University of Texas at Arlington, Arlington, TX USA

**Keywords:** Central Sensitization Inventory (CSI), Central sensitization, Central sensitivity syndrome, Chronic pain, Psychometrics, Spanish

## Abstract

**Purposing:**

The Central Sensitization Inventory (CSI) is a new patient-reported instrument, which measures symptoms related to Central Sensitivity Syndromes and Central Sensitization. The aim of this study was to translate the CSI into Spanish, and then to perform a psychometric validation, including a factor analysis to reveal the underlying structure.

**Methods:**

In this two-stage psychometric study participated 395 subjects with various chronic pain conditions and that were recruited from two Primary Care Centres. The CSI was cross-culturally adapted to Spanish through double forward and backward translations. The psychometric properties were then evaluated with analyses of construct validity, factor structure and internal consistency. One subgroup (n = 45) determined test-retest reliability at 7 days.

**Results:**

The Spanish Version of CSI demonstrated high internal consistency (α = 0.872) and test-retest reliability (r = 0.91). Factor structure was one-dimensional and supported construct validity.

**Conclusions:**

The psychometric properties of the Spanish version were found to be strong, with high test-retest reliability and internal consistency, with similar psychometric properties to the English language version. Unlike the English version, however, a one factor solution was found to be a best fit for the Spanish version.

## Background

Patient-reported outcome (PRO) measures (Garratt 2009) are commonly used to assess a patient’s symptoms or functional status. Although PRO are subjective, it can help clinicians better understand how a condition influences a patient’s capabilities or symptoms (Fayers and Machin 2013). Physical symptoms are often unexplained by a specific organic cause. In fact, no organic explanation can be found in 10 % of patients who report persisting physical symptoms (Rief et al. 2001). Furthermore, multiple somatic symptom occurrence is associated with higher rates of psychopathology and predict poorer treatment outcomes (Lydiard et al. 1993; Ahles et al. 1991). The phenomenon of central sensitization (CS) has been proposed to explain some incidents of “non-organic” symptoms. CS involves an abnormal increase of pain caused by neuronal hyperexcitability and dysfunction in descending and ascending pathways in the central nervous system (Kindler et al. 2011; Heinricher et al. 2009). Central sensitivity syndrome (CSS) is a proposed category of interrelated disorders, with a common etiology of CS (Kindler et al. 2011; Heinricher et al. 2009; Tracey and Dunckley 2004; Yunus 2000). Its family includes fibromyalgia, chronic fatigue syndrome, irritable bowel syndrome, temporomandibular joint disorder, and migraine/tension–type headache (Kindler et al. 2011; Heinricher et al. 2009; Tracey and Dunckley 2004; Yunus 2007).

The Central Sensitization Inventory (CSI) was designed as a tool to identify when a patient’s symptoms may be related to CS/CSSs (Neblett et al. 2015). Their identification ensures the most appropriate treatment, and may prevent inappropriate diagnostic testing. Part A of the CSI assesses 25 health-related symptoms common to CSSs, with total scores ranging from 0 to 100. Part B (which is not scored) asks if one has previously been diagnosed with one or more specific disorders, including seven separate CSSs. The original English version of the CSI was initially validated, having good psychometric properties (Neblett et al. 2015). Subsequent studies have found the CSI to be highly associated with the presence of CSS diagnoses in chronic pain patients (Neblett et al. 2013, 2015; Mayer et al. 2012). A score of “40” has been proposed as a cut-off score (Neblett et al. 2013, 2015; Mayer et al. 2012). More recently, CSI severity ranges have been proposed (Neblett et al. 2016).

Translations and validation studies of the CSI have been completed, or are currently proceeding, in a number of different languages, including Dutch (Kregel et al. 2015), French (Pitance et al. 2016) and others (personal communication). Therefore, the aim of the current study was to translate the CSI into European-style Spanish (CSI-Sp), and to subsequently validate the psychometric properties.

## Methods

A two-stage psychometric study was conducted. First, an initial translation and cross-cultural adaptation of the CSI, from English to Spanish, was performed. Then, a physical therapy outpatient population was used for evaluation of the CSI-Sp’s critical psychometric properties. The translation into Spanish was aimed to ensure conceptual equivalence of all of the test items, while maintaining proper cultural linguistic qualities. As detailed in the literature, a direct- and reverse-translation methodology was utilized by a specialist in the field (Cuesta-Vargas et al. 2010; Muñiz et al. 2013).

A total of 395 volunteers (54.4 ± 13.6 years, 55.6 % male) were recruited consecutively from the community-based Physiotherapy Program at the Malaga University. Exclusion criteria were; Chronic musculoskeletal pain for less than 3 months; diagnosis of specific medical conditions that can negatively affect the central nervous system, including cancer, brain or spinal cord injury, neurological disease or injury; Aged <18 years old and Poor Spanish language comprehension. Diagnoses were made by a physician in two primary care centres in Torremolinos, Malaga, Spanish National Health Service. All eligible participants completed the three Spanish language versions of the self-administered questionnaire CSI-Sp.

### Statistics

Descriptive analyses were applied to calculate means and standard deviations of demographic variables. Distribution and normality were determined by one-sample Kolmogorov–Smirnov tests (significance <0.05). Construct validity and factor structure were determined through the use of questionnaire principal component analysis with Maximum Likelihood Extraction (MLE), with the requirements for extraction being the satisfaction of all three points: screeplot inflection point, Eigen value >1.0 and accounting for >10 % of variance (Costello and Osborne 2005). The recommended minimum ratio of five participants-per-item was satisfied (Costello and Osborne 2005). Internal consistency of the scale items was determined from Cronbach’s α coefficients as calculated at an anticipated value range of 0.80–0.95 (Terwee et al. 2007; Cronbach 1951). Reliability was performed using the Intraclass Correlation Coefficients Type 2,1 (ICC_2.1_) test–retest methodology in a randomly selected subgroup of the full sample determined at 7 days (n = 45, 49 ± 5.2 years, 51.1 % female).

An error range of 0 ± 10 % was allowed in determining the test–retest reliability. The *MDC*_90_ analysis was performed as described by Stratford (2004). The standard error of the measurement (SEM) was calculated using the formula: SEM = s√(1 − r), where s = the mean and standard deviation (SD) of Time 1 and Time 2; r = the reliability coefficient for the test and Pearson’s correlation coefficient between test and retest values. Thereafter, the MDC_90_ was calculated using the formula: MDC_90_ = SEM × √2 × 1.96. All statistical analyses were conducted using the SPSS 21.0 for Windows. Ethical clearance was approved by the Tribunal of Review of Human Subjects at the University of Malaga.

## Results

The demographic and frequency of diagnoses of the sample are detailed in Table 1. The Spanish version of CSI provided can be found in “Appendix”. The normative values from CSI-Sp score were 24.6 ± 12.0 points (mean, SD). CSI-sp score distribution is detailed in Table 2.

**Table 1:** Anthropometric variables, CSI punctuation, most common diseases and diagnoses from CSI part B

*n* *=* *395*	
Avg age (SD)	55.07 (12.72)
Avg weight [(Kg) (SD)]	71.84 (14.05)
Avg height [(m) (SD)]	1.67 (0.09)
Avg BMI [(kg/m^2^) (SD)]	25.61 (4.16)
*Gender*	
Men [% (n)]	55.6 % (219)
Women [% (n)]	44.4 % (176)
Low back pain [% (n)]	55.2 % (216)
Neck pain [% (n)]	34.3 % (134)
Back pain [% (n)]	11.5 % (45)
Knee pain [% (n)]	6.6 % (26)
Artrhosis [% (n)]	5.4 % (21)
Shoulder pain [% (n)]	4.6 % (18)
*CSI part B*	
Restless leg syndrome [% (n)]	3.3 % (13)
Chronic fatigue syndrome [% (n)]	2.3 % (9)
Fibromyalgia [% (n)]	5.9 % (23)
TJD [% (n)]	6.4 % (25)
Migraine or tension headaches [% (n)]	11.8 % (46)
Irritable bowel syndrome [% (n)]	7.9 % (31)
Multiple chemical sensitivities [% (n)]	1 % (4)
Neck injury (including whiplash) [% (n)]	21 % (82)
Anxiety or panic attacks [% (n)]	11.8 % (46)
Depression [% (n)]	10.7 % (42)

Values expressed as mean (Standard Deviation) and percentage (n)

*BMI* Body Mass Index, *CSI* Central Sensitization Inventory, *TJD* temporomandibular joint disorder

**Table 2:** CSI-Sp score divided by punctuation <or> than 40 points (%, n) and scores (mean, SD) divided by main diagnostics

Diagnosis	CSI < 40 % (n)	CSI > 40 % (n)	CSI punctuation (mean, SD)
Low back pain	58.1 (165)	37.8 (14)	25.85 (11.21)
Neck pain	32.7 (93)	32.4 (12)	25.02 (10.25)
Back pain	12 (34)	13.5 (5)	23.80 (11.78)
Knee pain	6.3 (18)	8.1 (3)	24.42 (11.33)
Arthrosis	4.2 (12)	5.4 (2)	28.64 (15.82)
Shoulder pain	5.3 (15)	2.1 (7)	21.75 (10.14)

The CSI-Sp showed no missing responses and it showed a high degree of internal consistency (Cronbach’s α = 0.872) with an individual item range from 0.851 to 0.891. The test–retest reliability was high at (ICC_2.1_ = 0.91) with an individual range from 0.87 to 0.95. Measurement error was determined from SEM and MDC_90_, being at 2.52 and 7.83 %, respectively. No significance differences were found between genders in item responses.

**Fig. 1:**
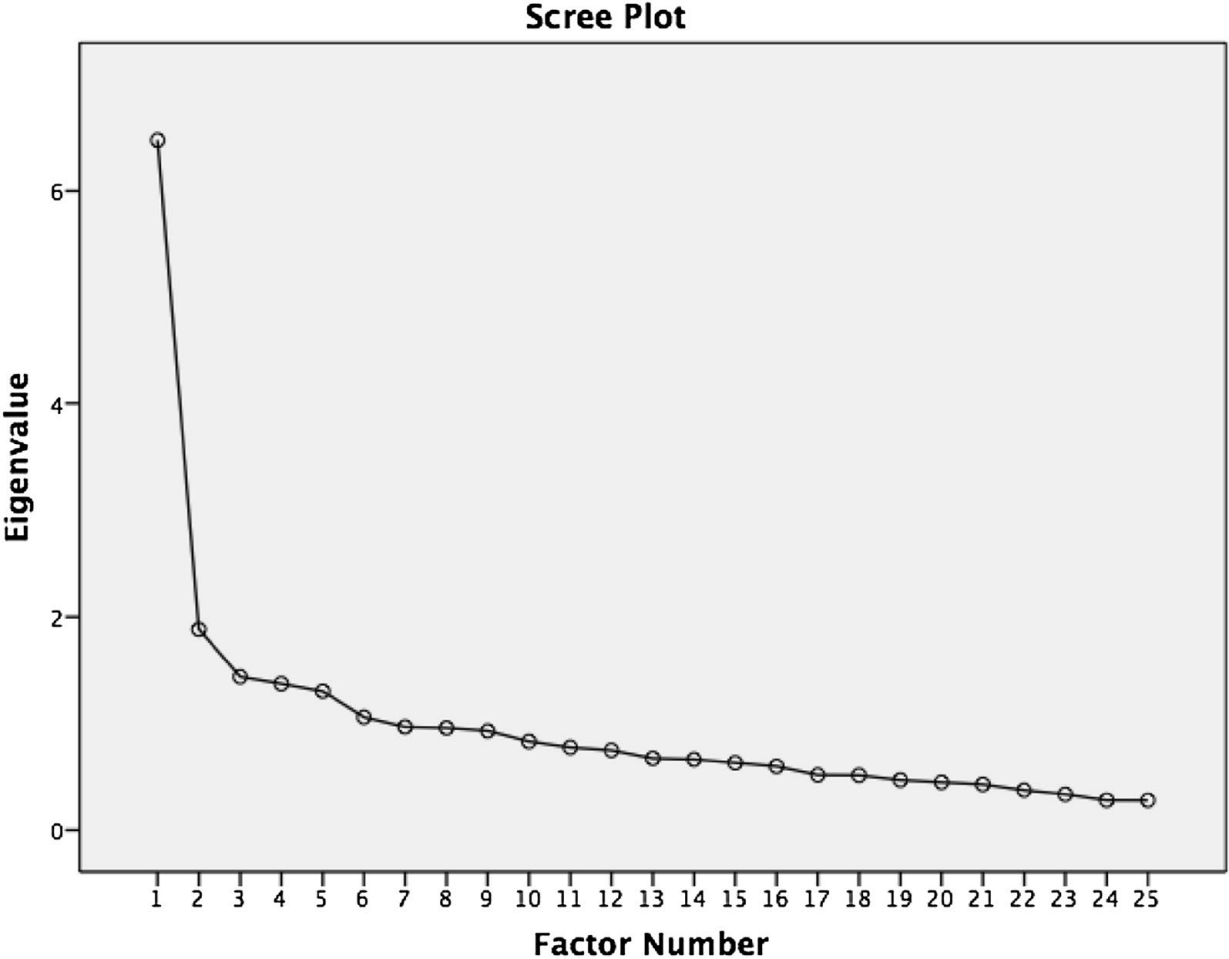
Scree plot indicating one factor solution

The correlation matrix for the CSI-Sp was determined suitable from the Kaiser-Meyer-Oklin values (0.864) and Barlett’s Test of Sphericity (*p* < 0.001). This indicated that the correlation matrix was unlikely to be an identity matrix and, therefore, was suitable for MLE. The factor analysis revealed a satisfactory percentage of total variance explained by the one factor at 25.9 %. However, the items with an Eigenvalue >1.0 each accounted for <10 % of variance and were shown to be after the screeplot initial inflection point and consequently not extracted. The screeplot (see Fig. 1) indicated a one-factor solution. The item loading for the one-factor solution for the MLE method and average score for each item is shown in Table 3. The Goodness-of-fit test revealed a Chi square of 866.04 (*p* < 0.000).

**Table 3:** Factor loading for each item after maximum likelihood extraction

	Factor
	1
Me siento cansado y desanimado cuando me levanto por las mañanas	0.612
Mis músculos están tensos y doloridos	0.650
Tengo ataques de pánico	0.388
Rechino los dientes o aprieto la mandíbula	0.342
Tengo problemas de diarrea o estreñimiento	0.400
Necesito ayuda pare realizar mis actividades diarias	0.476
Soy sensible a la luz brillante	0.440
Me canso fácilmente cuando estoy físicamente activo	0.724
Siento dolor en todo mi cuerpo	0.582
Tengo dolores de cabeza	416
Tengo molestia en mi vejiga o sensación de quemazón al orinar	0.294
No duermo bien	0.504
Tengo dificultad para concentrarme	0.436
Tengo problemas en la piel como resequedad, picor o sarpullido	0.299
El estrés hace que mis síntomas físicos empeoren	0.587
Me siento triste o deprimido	0.621
Me siento con poca energía	0.718
Tengo tensión muscular en mi cuello y hombros	0.555
Tengo dolor en mi mandíbula	0.426
Algunos olores, como perfumes, me hacen sentir náuseas.	0.269
Tengo que orinar frecuentemente	0.276
Mis piernas se sienten incómodas e inquietas cuando intento dormir por la noche	0.476
Tengo dificultad para recordar cosas	0.482
Sufrí algún trauma cuando era niño (a)	0.120
Tengo dolor en mi zona pélvica	0.289

## Discussion

In the present study, a cross-cultural adaptation of the CSI, from English to the Spanish, was completed, resulting in a CSI-Sp version of this Inventory. Construct validity and internal consistency of the CSI-Sp were determined independently, and were both found to be strong. The single factor structure from this psychometric properties indicated that a single summated score could be used (Doward and McKenna 2004). The one-factor solution that emerged in the factor analysis accounted for a significant proportion of variance, and showed evidence supporting the presence of construct validity. The findings of the current study, however, is contrast with the English (Mayer et al. 2012), Dutch (Kregel et al. 2015), and French (Pitance et al. 2016) versions. The first two versions revealed a 4-factor model, and the French version produced 5-factors. However these studies did not satisfy the three point requirements for extraction, as recommended by Costello and Osborne 2005 and in the other hand, our study shown a low variance explained (Costello and Osborne 2005). Both English and Dutch versions demonstrated 3 (Garratt 2009; Heinricher et al. 2009; Costello and Osborne 2005; Terwee et al. 2007) or 5 (Garratt 2009; Heinricher et al. 2009; Kregel et al. 2015; Stratford 2004) items with an insufficient load on any factor. A one-factor solution is critical if a PRO is used with a single summated score, and it subsequently reflects the construct for which it is primary employed—that of representation of the only CS condition.

High test–retest reliability, was found (ICC = 0.91), which was in-line with the test–retest results of the English (0.82) (Mayer et al. 2012), Dutch (0.88) (Kregel et al. 2015) and French versions (0.91–0.94) (Pitance et al. 2016). Consequently, the current study shows that the CSI-Sp should prove to be a reliable instrument. Internal consistency analysis showed a level of 0.872, below the accepted 0.95 thresholds for item redundancy (Terwee et al. 2007). Similarities were found in the internal consistency of all 25 items of the CSI in the original study of the English version (Cronbach’s α = 0.879) (Mayer et al. 2012) and the Dutch version (Kregel et al. 2015) (Cronbach’s α = 0.91).

This present translation proportionated accessibility to the CSI-Sp for the second largest geographically-used language (United Nations 2016) A cross-cultural adaptation of a scale has been previously done to be applied in the Spanish context (Muñiz et al. 2013). It is critical to employ valid and reliable research measures which are culturally and linguistically appropriated.

The strengths of the present study included its prospective nature and adequate number of subjects; the inclusion of consecutive patients; and the limited selection bias (Kass and Tinsley 1979). Obtaining results supporting the psychometric properties of the previous research on the original English version indicates that may it be possible to compare Spanish and English population and that cross-cultural adaptions would be appropriate to other diverse linguistic groups.

One limitation of the present study is the lack of longitudinal data regarding other psychometric properties and not including Hispanic/Latino/South American participants, which would have potentially provided confirming or conflicting linguistic information. Hence, it would be appropriate to include them in futures studies. Other limitation was the sample size to run Confirmatory Factor Analysis focussing to identify the best factor structure, in this way we are started a pool of data (n > 2000) across the different countries/languages (US, Spain, Belgium, France, Serbia, Italy and Brazil).

## Conclusions

The psychometric properties of the CSI-Sp are reported for the first time. The determined values were satisfactory and supportive of the validation of the CSI-Sp, particularly in the areas of internal consistency, factor structure and reliability. Consequently, the CSI-Sp may be useful in Spanish-speaking populations and for making cross-cultural comparisons in other English-speaking countries with a high Spanish-speaking population.
